# National initiatives to improve outcomes from out-of-hospital cardiac arrest in England

**DOI:** 10.1136/emermed-2015-204847

**Published:** 2015-09-23

**Authors:** Gavin D Perkins, Andrew S Lockey, Mark A de Belder, Fionna Moore, Peter Weissberg, Huon Gray

**Affiliations:** 1 Out of Hospital Cardiac Arrest Outcomes, Warwick Clinical Trials Unit, University of Warwick, Coventry, UK; 2 Resuscitation Council (UK), Tavistock House North, Tavistock Square, London, UK; 3 National Institute for Cardiovascular Outcomes Research (NICOR), UCL Institute of Cardiovascular Science, London, UK; 4 National Ambulance Services Medical Directors’ Group, London Ambulance Service NHS Trust, London, UK; 5 British Heart Foundation, Greater London House, London, UK; 6 National Clinical Director (Cardiac), NHS England, University Hospital Southampton, Southampton, UK

**Keywords:** cardiac arrest, arrythmia, cardiac care, treatment, emergency ambulance systems, resuscitation

NHS England report that the ambulance services attempt to resuscitate approximately 28 000 people from out-of-hospital cardiac arrest each year (approximately 1 per 2000 inhabitants per year).^1^ The rate of initial success (return of spontaneous circulation) was 25%, with less than half of those who are successfully resuscitated initially surviving to go home from hospital (survival to discharge 7%–8%, 2011–2014).^1^ (see figure 1). The survival rates contrast sharply with those observed in the best-performing emergency medical services systems, which have survival rates of 20%–25%.^2–4^ In 2013, the government's Cardiovascular Disease Outcomes Strategy for England set the ambitious, but achievable target of increasing survival from out-of-hospital cardiac arrest by 50%, leading to an additional 1000 lives saved each year.

**Figure 1:**
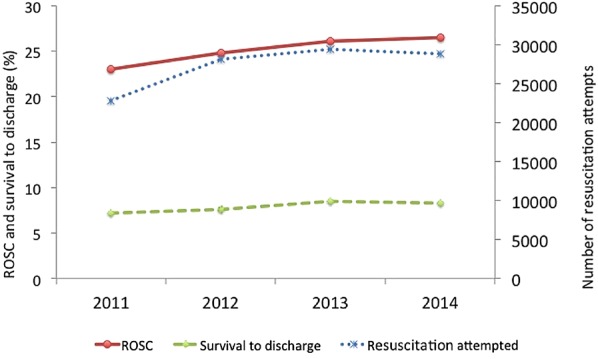
Summary of number of resuscitation attempts, return of spontaneous circulation (ROSC) and survival to discharge.

## The chain of survival

Improving outcomes from cardiac arrest requires improvements in one or more links in the chain of survival.^5^ The first link is early access. This step prioritises calling for help early in patients at risk of cardiac arrest (eg, those with chest pain) and those with signs of cardiac arrest (unresponsive and not breathing normally). An early response may allow cardiac arrest to be prevented or ensures trained staff arrive early to initiate/continue resuscitation. The second link highlights the critical importance of the bystander providing early cardiopulmonary resuscitation (CPR). Evidence from observational studies suggests that survival from cardiac arrest can be increased from twofold to fourfold with bystander CPR.^6–8^ Early defibrillation forms the third link as defibrillation within 3–5 min can produce survival rates as high as 50%–70%.^9–11^ The final link in the chain is early ALS and standardised postresuscitation care. These interventions are initiated by the emergency services, and continued during and after transfer of care to the hospitals.

Different strategies are needed to target the various elements of the chain of survival, and numerous projects have been initiated in the UK in past decade to tackle these issues.

The Cardiovascular Disease Outcomes strategy specifically relates to improving the performance of the middle two links, namely improving the rate of bystander CPR and the use of automated external defibrillators (AEDs). These are perceived to be the two weakest links in England at present. Evidence from Scandinavia and some parts of the USA has shown that targeting these areas can prove to be the most impactful in terms of improving survival rates.^2–4^ It is hoped that increasing the number of bystanders trained in CPR will also positively impact on the first link (early call for help) and the fourth link (ALS and postresuscitation care). ‘Working in partnership with the Resuscitation Council (UK) and British Heart Foundation the NHS Commissioning Board aims to improve the rate of bystander CPR and use of automated external defibrillators.’

The Cardiovascular Disease Outcomes strategy provided the opportunity to draw together the various stakeholders involved with ongoing projects under the auspices of NHS England to encourage further development and the sharing of good practice.^12^ This forum, called the Community Resuscitation Group, met for the first time in 2013 and identified the following as its aspirations:

- Establish CPR training in all schools and mobilise relevant organisations to ensure this is done.
- Identify simple consistent messages for the public, and achieve greater public awareness of what to do when faced with an out-of-hospital cardiac arrest.
- Improve audit and set up a national defibrillator database.
- Achieve a collaborative approach among stakeholders, including industry.
- Ensure that all activities are evidence based and where evidence is lacking, call for appropriate research.

## Improving bystander CPR

The time to initiating CPR in cardiac arrest is critical to outcome. Even the highest-performing ambulance systems will be unable to get to a patient and start CPR more rapidly than a bystander who is present at the scene of the cardiac arrest. Therefore, any strategy to improve outcomes from cardiac arrest must seek to improve the bystander response to cardiac arrest.

Despite the clear benefits of bystander CPR, early data from the Out of Hospital Cardiac Arrest Outcomes project (http://www.warwick.ac.uk/go/ohcao) indicates that CPR is undertaken on average in only 40–50% of cardiac arrests. This figure is substantially lower than figures published for countries with comparable demographics (bystander CPR rate in Norway is 73%,^2^ Seattle 66%,^3^ North Holland 60%^4^), and may, in part, explain why current (2011–2014) survival rates from out-of-hospital cardiac arrest in the UK lags behind that of other countries (Norway 25%,^2^ Seattle 22%,^3^ Holland 21%^4^).

Several, often multifaceted, approaches have proved to be successful in improving bystander CPR rates. CPR training delivered through mass training events,^13^ targeting family members of patients at risk of cardiac arrest,^13^ mandatory CPR testing as part of driving licence qualification,^5^ ^14^ ^15^ community CPR training^13^ and CPR training in schools^2^ ^5^ have been successful. Other promising modalities include video/DVD training, ultra-brief CPR training and mobile phone apps.^16^ ^17^

Through the Heartstart programme, the British Heart Foundation has invested in CPR training for the lay public since the 1990s. In 2010, the BHF and Resuscitation Council (UK) (RCUK) initiated a campaign to raise awareness of out-of-hospital cardiac arrest, and the importance and effectiveness of bystander CPR.^18^ The campaign involves several facets, including supporting CPR training in schools and communities, production of the Lifesaver app and national media campaigns. The knowledge that brief and ultra-brief hands-only CPR training videos increase the likelihood that a bystander will attempt CPR and lead to superior CPR skills compared with untrained laypersons^19^ informed the BHF Vinnie Jones Hands-only CPR campaign and the Hands Only CPR app.^20^ The award-winning Lifesaver app^21^ was developed by the RCUK, and has been distributed free of charge to all schools in the UK. Lifesaver is a live-action movie that you play like a game. It throws you into the heart of the action, changing what happens in three scenarios showing real people in real places. Several cases of successful resuscitation from bystanders trained with these tools confirm the potential of this approach.

In 2014, the BHF launched its Nation of Lifesavers campaign to coincide with European Re-start a Heart Day on 16th October. This campaign seeks to ensure that large proportions of the UK population are skilled and equipped to start the chain of survival immediately in the event of an out-of-hospital cardiac arrest. To achieve this, BHF launched a DVD-based, watch-and-learn model of CPR training to secondary schools, community groups and workplaces across the UK, under the banner of ‘Call, Push, Rescue’ to give a more lay-friendly descriptor than ‘cardiopulmonary resuscitation’. The aim of the training is to demystify CPR and reinforce the message that it can be learnt and delivered by anyone so that members of the public are more likely to intervene in the event of an out-of-hospital cardiac arrest.

BHF has developed a marketing strategy to secondary schools in particular (to whom the training packs are provided free of charge), which celebrates the lives saved by members of the public who carried out CPR on loved ones and strangers. In addition, several thousand people have already learned CPR at work using the Call Push Rescue model. BHF is actively working with some of the UK’s largest corporate employers who have agreed to purchase the Call Push Rescue CPR training kit so that their staff can learn CPR in less than 30 min.

In March 2012, the Bolton Wanderers footballer Fabrice Muamba suddenly collapsed on the pitch during a game, and was successfully resuscitated. He has supported the work of the BHF and RCUK. In addition, he has supported the ‘Hearts and Goals’ campaign that was set up by the Arrhythmia Alliance after Bolton Wanderers chose them as their national charity.

The primary goal from the various campaigns and strategies has been to ensure that every young person leaves school knowing how to perform CPR and having an awareness of the use of a public access defibrillator.^22^ This has been achieved in other countries by delivering this training as part of a national curriculum. Although this is yet to be achieved, there have been several notable successes in the 5 years that the campaigns have been active.

1. Increased public awareness has undoubtedly led to an increased number of schools delivering this training. In some regions (eg, Leicestershire, North West, London), there has been a regional approach to coordinating training.
2. Successful lobbying of the European Parliament to pass a Written Declaration in 2012 supporting the development of national strategies to improve bystander CPR rates and access to defibrillators.
3. Development of guidance about purchase of defibrillators for schools in England by the Department for Education.^23^

Looking forward, the Community Resuscitation Group intends to continue the call for all school children to be taught CPR. The annual Restart a Heart day on 16 October will once again showcase innovative strategies from around the country to improve CPR rates, and it is essential that all of this good practice is shared. The ongoing work for the next year will also involve analysing a system-wide approach to community resuscitation and the subsequent development of national guidance that encapsulates best recommended practice.

## Improving bystander defibrillation

Early defibrillation for patients in ventricular fibrillation or pulse-less ventricular tachycardia is critical since for every minute defibrillation is delayed, the chances of survival falls by approximately 10%.^24^ ^25^ The development of AEDs and their placement in public places open up exciting opportunities to shorten the time from collapse to first shock.^26^ From April 2000 to November 2002, the Department of Health (England) placed 681 AEDs in 110 public places for use by volunteer lay first responders.^27^ The Public Access Defibrillation (PAD) trial confirmed that the implementation of PAD programmes could double survival from cardiac arrest.^28^ However, for such programmes to achieve their full potential requires an effective community response.^29^

Recent UK data reported bystander defibrillation rates of only 1.74% of out-of-hospital cardiac arrest (OHCA) cases.^30^ ^31^ Potential explanations for the low use of bystander-assisted defibrillation include bystanders lacking confidence about the location of and how to use the devices. In the Netherlands, a co-ordinated strategy designed to decreasing the time to first shock delivery by more widespread use of the AED by dispatched rescuers (firefighter/police team) and by layperson rescuers using publicly available AEDs saw overall survival improve from 16.2% to 19.7% and from 29.1% to 41.4% among those with initially shockable rhythms. Just as ambulance-dispatcher-assisted CPR has been shown to improve uptake of CPR,^32^ it is likely that if a system existed for emergency ambulance services to locate and send bystanders to bring the nearest AEDs to the patient, rates of AED use and survival would increase.

To facilitate such a system in the UK, the BHF is scoping the feasibility of developing a national database, which would be made available in real time to ambulance services, allowing the emergency medical dispatchers to direct members of the public to the nearest PAD. Integrating such a database with mobile phone technology may assist with the initial locating of AEDs,^33^ and facilitate their subsequent deployment including provision of on-line user support.^34^ At present, there is no single national database and the challenges of setting one up, including the ability to capture all defibrillators (both publically and privately owned), should not be underestimated. This has been the primary focus for the Community Resuscitation Group over the last 2 years, and it is hoped that the successful establishment of a national database will significantly improve survival rates.

Various stakeholders in the Community Resuscitation Group were integrally involved in the development of the ‘Automated External Defibrillator: Guide for Schools’ by the Department for Education in 2014.^23^ An associated tendering process led to discounted AEDs being made available to schools to purchase. By 17 April 2015, 360 AEDs had been purchased by schools through this scheme.

Finally, the Group is reviewing the signage used for public access defibrillators. There is a fear that the current sign with a prominent lightning bolt may deter some rescuers from using them. Various design concepts are being tested with the public.

## Continuous quality improvement

Ongoing, systematic collection and analysis of data about out-of-hospital cardiac arrest and bystander CPR is essential to the planning, implementation and evaluation of effective CPR programmes. The BHF and RCUK established a national out-of-hospital cardiac arrest outcomes (OHCAO) registry in partnership with the National Ambulance Service Medical Directors Group and University of Warwick. The OHCAO registry (http://www.warwick.ac.uk/go/ohcao)^35^ collects process and outcome information based on the international Utstein template^36^ about patients who are treated by ambulance services for cardiac arrest. The registry will provide a tool to support local quality-improvement initiatives, and will facilitate measuring the impact of the interventions described above. Although participation in the registry is voluntary, it is strongly endorsed by the National Ambulance Service Medical Directors Group.

The BHF recently hosted a meeting of the various professional societies that have an interest in improving outcomes for patients with out-of-hospital cardiac arrest, as well as reviewing the various data collection systems in place and how they might be best used. A work stream will be developed with the aim of achieving a consistent clinical pathway for these patients that maximises the chance of a good recovery.

When evaluating outcome from OHCA, one needs to be careful about the measures considered. In the UK, emergency ambulance services attend approximately 60 000 cases of suspected cardiac arrest each year, but resuscitation is only appropriate, and undertaken, in approximately 45% of cases.^37^ ^38^ This is because in some cases the victim has been dead for several hours, or has suffered severe trauma, which is not compatible with life, or because the opportunity to start resuscitation was not taken sooner, while the emergency medicinal services (EMS) were on their way. If more bystanders had the confidence and skills to call for an ambulance quickly, deliver effective CPR until the EMS arrive and when appropriate use a public access defibrillator, the number of cases where the EMS could attempt resuscitation would increase. Therefore, when evaluating the impact of a programme, one must consider the survival rates and the total number of cardiac arrest survivors per head of the population per year.

## Learning together

Cardiac arrest is a global problem, and communities around the world can learn from each other’s approaches. Integrated system-wide approaches have shown success in a number of settings. In North Carolina, the HeartRescue project comprising community CPR training, AED deployment and dispatcher-assisted CPR improved bystander responses and survival.^13^ Denmark's approach included CPR in schools’ training, distribution of CPR self instruction kits and a mandatory resuscitation course when acquiring a driver's licence worked in combination to increase survival rates from 3% to 10%.^5^ Sharing information about the successes and failures of different initiatives is critical to enabling progress. We commend the Institute of Medicine recent report, which has several parallels with our approach (surveillance, community response, education and training) and also champions the importance of research to identify new treatments and approaches, translation of research into practice and continuous quality improvement. We hope such global efforts will have a sustained impact on outcome from cardiac arrest.

## Summary

Since 2010, the RCUK and BHF have developed a suite of interventions designed to strengthen the community response to cardiac arrest intended to lead to more people surviving from cardiac arrest. The establishment of the Community Resuscitation Group in 2013, under the auspices of NHS England, has brought together an even wider range of stakeholders to build on this work and develop further initiatives.

Rigorous evaluation of these initiatives is required to ensure that they achieve the expected number of lives saved. The establishment of a national registry will play a key role in that evaluation and in identifying areas for future quality-improvement work.
